# Assessing the Reliability of Arterial Blood Gas Analyzer for Sodium and Potassium Measurement in Critical Care Management

**DOI:** 10.1155/bmri/9203768

**Published:** 2026-06-25

**Authors:** Madhusha Gonapaladeniya, Indika Siriwardana, Bolonghoge Dayanath, Harith Wickramasekara

**Affiliations:** ^1^ Department of Medical Laboratory Sciences, Faculty of Allied Health Sciences, University of Sri Jayewardenepura, Nugegoda, Sri Lanka, sjp.ac.lk; ^2^ Department of Biochemistry and Clinical Chemistry, Faculty of Medicine, University of Moratuwa, Moratuwa, Sri Lanka, mrt.ac.lk; ^3^ Department of Pathology, North Colombo Teaching Hospital, Ragama, Sri Lanka, health.gov.lk; ^4^ Department of Medical Education, Faculty of Medicine, University of Kelaniya, Kelaniya, Sri Lanka, kln.ac.lk

**Keywords:** arterial, blood, critical care, electrolytes, gas

## Abstract

Critically ill patients often experience fluid and electrolyte imbalances, often due to medical conditions or medications. Therefore, evaluating serum electrolyte levels is crucial in managing these patients. This study is aimed at assessing the arterial blood gas analyzer′s accuracy in measuring serum electrolytes at the Emergency Treatment Unit (ETU) of Teaching Hospital Karapitiya (THK), Galle. This cross‐sectional study was conducted with 67 ETU, THK, patients, and their sodium (Na^+^) and potassium (K^+^) levels were measured using both ABG and ISE analyzers. The study examined the correlation between the two methods for Na^+^ and K^+^ and assessed the validity of the ABG analyzer through *t*‐tests. Additionally, the clinical significance of these methods was evaluated using Cohen′s *d* effect size (ES) values. The mean Na^+^ value was 130.87 ± 9.522 mmol/L for the ABG analyzer and 130.30 ± 8.753 mmol/L for the ISE analyzer. The K^+^ values were 3.80 ± 1.055 and 3.93 ± 0.835 mmol/L, respectively. The correlation coefficients between the two methods were 0.875 for Na^+^ values and 0.905 for K^+^ values. *p* values were 0.275 and 0.024, respectively. The ES value for Na^+^ values measured using ABG and ISE was 0.062, and for K^+^, it was 0.157. The Na^+^ concentrations exhibited no statistically significant difference, while K^+^ concentrations demonstrated statistical significance. However, the observed disparities in the concentrations of both parameters were of limited clinical significance. Clinicians can confidently rely on the ABG analyzer as a dependable tool for measuring Na^+^ and K^+^ values in the management of critically ill patients.

## 1. Introduction

An electrolyte is a substance that ionizes when dissolved in suitable ionizing solvents such as water. Electrolytes are minerals in human blood and other body fluids that carry an electric charge [1]. Serum electrolyte levels are altered in many disease conditions. Renal diseases are a common condition. This is mostly seen in patients who go through dialysis. Glomerular nephritis also affects renal disease [2]. Electrolyte levels may be altered in cardiovascular emergencies [3]. Therefore, a serum electrolyte panel is regularly ordered in the diagnosis of ICU patients. Studies had been conducted earlier to assess the significance of serum electrolytes in managing emergency patients [4] and pediatric patients [5]. Clinicians always find interest in the altered physiological basis of serum electrolyte concentrations in managing ICU patients. Hyponatremia is the reduction of sodium (Na^+^) value < 136 mmol/L, whereas a serum Na^+^ concentration > 145 mmol/L is called hypernatremia. Serum potassium (K^+^) levels < 3.5 and > 5 mmol/L are defined as hypokalemia and hyperkalemia, respectively. Identification of true causes for these imbalances is clinically significant [6].

Patients receiving intravenous fluids are monitored during short periods, as small fluctuations in serum electrolyte levels are significant in managing these patients. Validations using different methods, such as direct ISE and indirect ISE, in these types of cases are important [7].

Different analytical methods, such as flame atomic emission spectrometry, ISE, and enzymatic methods, have been used in studies to measure serum electrolyte levels in blood [8, 9]. However, when assessing critical patients, the speed of obtaining biochemical investigation results is as vital as the accuracy of the test results. In previous studies, researchers have measured serum electrolytes using point‐of‐care blood gas and automated analyzers in central laboratories [10, 11]. Electrolyte concentrations can be measured using either direct ISE or indirect ISE methods. Direct ISE is used in blood gas analyzers and most other point‐of‐care testing (POCT) devices, whereas indirect ISE is used in the majority of central laboratory biochemical analyzers [12].

During the study, a comparison was performed between the ABG analyzer and the central laboratory ISE analyzer. Although comparisons had been made in the past in different setups, no such comparisons have been made in Sri Lanka, according to the best of our knowledge. Also, the previous work in this area did not come to a common conclusion about the validity of ABG in measuring serum electrolytes.

## 2. Materials and Methods

This cross‐sectional study was performed to assess the comparability of serum electrolyte concentrations measured in ABG and ISE analyzers. The Emergency Treatment Unit (ETU) of Teaching Hospital Karapitiya (THK) was selected as the study setting, as critically ill patients from different parts of the southern province are admitted to the ETU, THK. A sample of 67 patients who were admitted to the ETU, THK, was selected for this study.

### 2.1. Inclusion and Exclusion Criteria

Patients admitted to the ETU, THK, who were undergoing ABG analysis, were included in this study. Only adult patients (≥ 18 years of age) were recruited for this study. Pediatric patients (< 18 years of age) were excluded from the study. Other exclusion criteria included patients with known hemolysis, lipemia, or those who declined consent.

Ethical approval was obtained from the Ethics Committee of the Faculty of Medicine, University of Ruhuna.

### 2.2. Sample Collection and Processing

Arterial blood samples were collected from the radial artery using the standard arterial puncture technique. The samples were drawn into heparinized syringes (containing lithium heparin as the anticoagulant) specifically designed for arterial blood gas analysis, following the manufacturer′s specifications for the GEM Premier 3000 analyzer. Appropriate heparinization was ensured to prevent sample dilution effects that could compromise result accuracy.

No additional blood samples were collected beyond the clinical requirement. The same arterial blood sample was used for both ABG and ISE analyses. After performing ABG analysis using the GEM Premier 3000 analyzer, the remaining blood from the same arterial sample was immediately transferred to a plain tube and used for ISE analysis in this study. The use of the same arterial blood sample for both methods eliminated the variability that might arise from different collection sites (arterial vs. venous) or different time points.

The result obtained from the ABG analysis was recorded. There was no delay in sample processing. Samples were centrifuged at 1500 g for 5 min, and plasma was analyzed in the SmartLyte ISE Analyzer in the central laboratory. Each sample was assayed for the two parameters of Na^+^ and K^+^ using the ISE analyzer. The assays were performed according to the manufacturer′s instructions. All parameters were measured in units of millimoles per liter.

The ISE analyzer (SmartLyte Analyzer) method was traceable to the Roche 9180 analyzer, and the ABG analyzer (GEM Premier 3000) was evaluated to meet US regulations with Instrumentation Laboratory, where Intelligent Quality Management (iQM) was practiced. Internal QC samples were analyzed in parallel with the test samples. QC pathological and normal were both used for validation. Violations of QC rules were monitored, and corrective actions were taken accordingly.

### 2.3. Statistical Analysis

The data were analyzed using SPSS 17.0 for the Windows operating system. Descriptive analysis and bivariate analysis were carried out. Pearson correlation coefficient and paired *t*‐test were used to analyze the data statistically. The clinical significance of the methods was assessed using Cohen′s *d* effect size (ES) values.

Additionally, Bland–Altman analysis was performed to assess agreement between the two measurement methods. Mean bias (average difference between ABG and ISE measurements) and 95% limits of agreement (LoAs) were calculated using the following formula: mean difference ± 1.96 × SD of differences. The LoA defines the range within which 95% of differences between the two methods are expected to fall. Clinically acceptable limits were defined based on biological variation criteria for Na^+^ (±4 mmol/L) and K^+^ (±0.3 mmol/L).

## 3. Results

A total of 67 patients admitted to the ETU, THK, were included in this study. Na^+^ and K^+^ values obtained from ABG analysis and ISE analysis were reviewed for the assessment of the validity of the ABG analyzer for serum electrolyte concentration measurement. The mean values of Na^+^ were 130.87 and 130.30 mmol/L for the ABG analyzer and for the ISE analyzer, respectively. The mean K^+^ values were 3.80 and 3.95 mmol/L, respectively. The SD values of Na^+^ were 9.522 and 8.753 for the ABG analyzer and ISE analyzer, respectively. SD values of K^+^ were 1.055 and 0.835, respectively.

The distribution of Na^+^ values revealed a pronounced skewness toward hyponatremia, with 76.1% of patients presenting with Na^+^ concentrations < 136 mmol/L. This distribution reflects the clinical reality of our ETU setting, where hyponatremic patients are commonly encountered.

When grouping the K^+^ values, similar results were observed, as given in Table 1. The counts and percentages were the same for the ABG analyzer and ISE analyzer under each class. Hyperkalemia cases were 6/67 (8.96%), normokalemia 31/67 (46.27%), and hypokalemia 30/67 (44.78%).

**Table 1:** Classification of sodium values.

Clinical condition	Count ABG‐Na^+^	% ABG‐Na^+^	Count ISE‐Na^+^	% ISE‐Na^+^	*M* *e* *a* *n* ± *S* *D*ABG‐Na^+^ (mmol/L)	*M* *e* *a* *n* ± *S* *D*ISE‐Na^+^ (mmol/L)
Hypernatremia	2	2.99	3	4.48	157.00 ± 0.00	149.00 ± 2.00
Hyponatremia	52	77.61	54	80.60	127.69 ± 7.78	127.65 ± 7.31
Normonatremia	13	19.40	10	14.93	139.54 ± 1.56	139.00 ± 1.94
Total	67	100	67	100	130.87 ± 9.52	130.30 ± 8.75

When the Na^+^ values of both the ABG analyzer and ISE analyzer were grouped into clinically significant classes, it revealed similar counts and percentages for the two methods under each group (Table 2). There were 2/67 (2.99%) for hypernatremia, 14/67 (20.90%) for normonatremia, and 51/67 (76.12%) for hyponatremia.

**Table 2:** Classification of potassium values.

Clinical condition	Count ABG‐K^+^	% ABG‐K^+^	Count ISE‐K^+^	% ISE‐K^+^	*M* *e* *a* *n* ± *S* *D*ABG‐K^+^ (mmol/L)	*M* *e* *a* *n* ± *S* *D*ISE‐K^+^ (mmol/L)
Hyperkalemia	6	8.96	7	10.45	6.33 ± 0.77	5.64 ± 0.47
Hypokalemia	34	50.75	23	34.33	3.06 ± 0.41	3.13 ± 0.30
Normokalemia	27	40.30	37	55.22	4.16 ± 0.41	4.10 ± 0.41
Total	67	100	67	100	3.80 ± 1.06	3.93 ± 0.83

### 3.1. Validity of ABG Analyzer in Measuring Serum Electrolytes

#### 3.1.1. Pair 1

The null hypothesis (H_0_): There was no significant difference in the two methods (ABG analysis and ISE analysis) used to measure Na^+^ values. The alternative hypothesis (H_1_): There was a significant difference in the two methods (ABG analysis and ISE analysis) which were used to measure Na^+^ values.

The *p* value of 0.275 was higher than *p* = 0.05 (*p* > 0.05) at a 95% confidence interval. Thereby, the null hypothesis was accepted. ABG‐Na^+^ values and ISE‐Na^+^ values showed no statistically significant difference between the two methods used. The mean difference between methods was 0.57 mmol/L (95% CI: −0.44 to 1.58 mmol/L).

#### 3.1.2. Pair 2

The null hypothesis (H_0_): There was no significant difference in the two methods (ABG analysis and ISE analysis) which were used to measure K^+^ values. The alternative hypothesis (H_1_): There was a significant difference in the two methods (ABG analysis and ISE analysis) used to measure K^+^ values.

ABG‐K^+^ values and ISE‐K^+^ values resulted in *p* = 0.024, which was less than 0.05 (*p* < 0.05). Thereby, the null hypothesis was rejected. There was a statistically significant difference between the K^+^ values obtained using the two methods at a 95% confidence interval. The mean difference between methods was −0.13 mmol/L (95% CI: −0.24 to −0.02 mmol/L).

The clinical significance of the methods was assessed using Cohen′s *d* ES values. The ES value for Na^+^ measured using ABG and ISE was 0.062, and for K^+^, it was 0.157. Both values were below 0.2, which suggested a low clinical significance.

**Figure 1:**
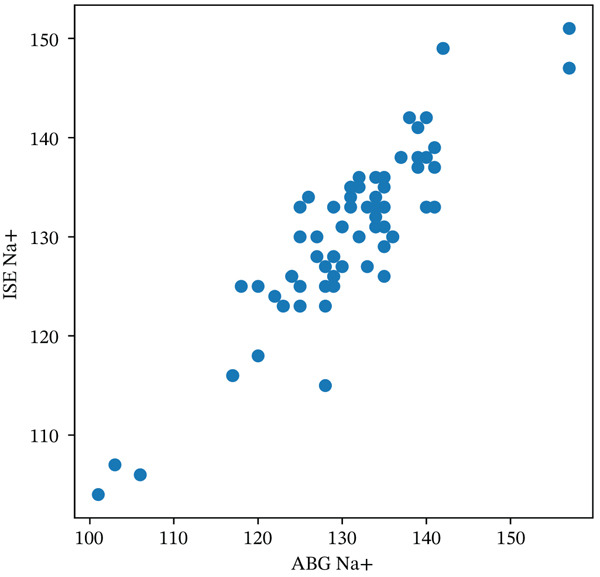
Scatter plot of ABG and ISE sodium values.

**Figure 2:**
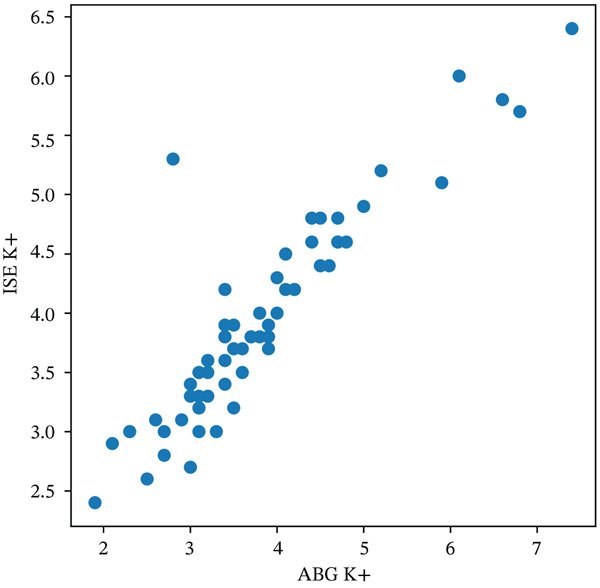
Scatter plot of ABG and ISE potassium values.

When considering the correlations, a positive correlation was observed between the results of the ABG‐Na^+^ concentrations and ISE‐Na^+^ concentrations with a correlation coefficient (*r*) of 0.897 and *r* ^2^ = 0.805 (*n* = 67, *p* < 0.01) (Figure 1). ABG‐K^+^ values and ISE‐K^+^ values also have a positive correlation with a correlation coefficient (*r*) of 0.905 (*r* ^2^ = 0.819) (*n* = 67, *p* < 0.01) (Figure 2).

### 3.2. Bland–Altman Agreement Analysis

**Figure 3:**
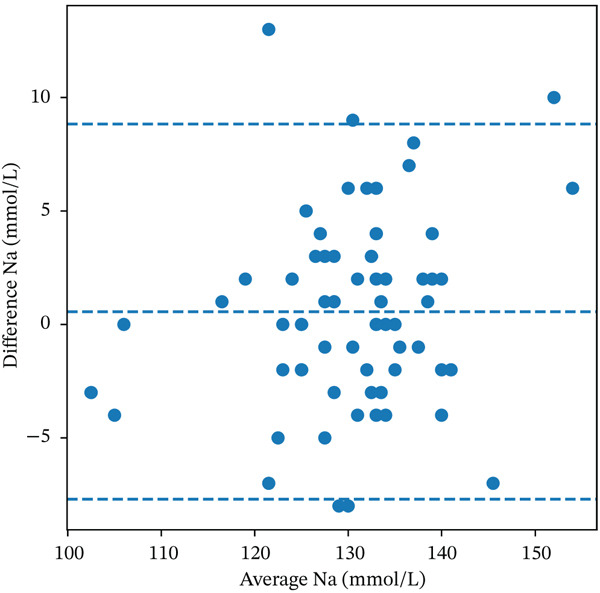
Bland–Altman plot showing agreement between ABG and ISE methods for sodium measurement. The figure shows the difference (ABG − ISE) on the *y*‐axis versus the mean of (ABG + ISE)/2 on the *x*‐axis. The solid horizontal line represents the mean bias (0.57 mmol/L), and the dashed lines represent the 95% LoAs (−7.69 to 8.83 mmol/L).

**Figure 4:**
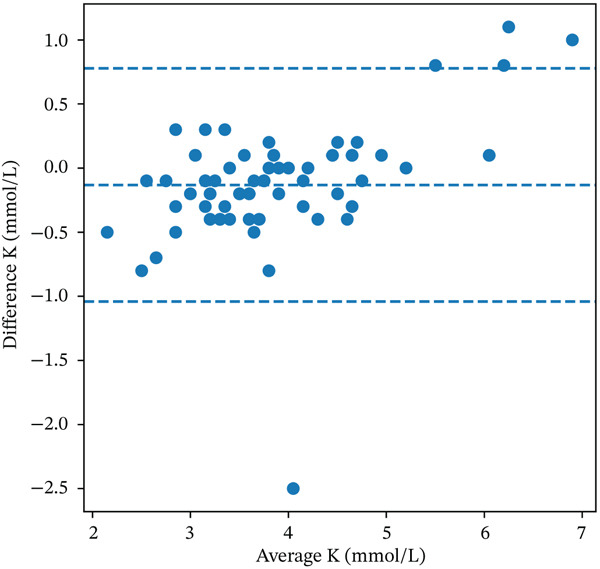
Bland–Altman plot showing agreement between ABG and ISE methods for potassium measurement. The figure shows the difference (ABG − ISE) on the *y*‐axis versus the mean of (ABG + ISE)/2 on the *x*‐axis. The solid horizontal line represents the mean bias (−0.13 mmol/L), and the dashed lines represent the 95% LoAs (−1.04 to 0.78 mmol/L).

Bland–Altman analysis was performed to assess the agreement between ABG and ISE methods (Figures 3 and 4).

For Na^+^: The mean bias (ABG minus ISE) was 0.57 mmol/L, indicating that ABG values were slightly higher than ISE values on average. The 95% LoAs ranged from −7.70 to 8.84 mmol/L. The majority of differences fell within ±8 mmol/L, which is within acceptable clinical limits for Na^+^ measurement. The bias was minimal and clinically insignificant.

For K^+^: The mean bias (ABG minus ISE) was −0.13 mmol/L, indicating that ABG values were slightly lower than ISE values on average. The 95% LoAs ranged from −1.04 to 0.78 mmol/L. All differences fell within ±1 mmol/L, which is clinically acceptable for K^+^ measurement. Despite the statistical significance of the difference (*p* = 0.024), the Bland–Altman plot demonstrates that the bias is small and clinically acceptable.

## 4. Discussion

POCT devices have gained significant traction in critical care settings, primarily due to rapid turnaround times. In the ETU at THK, validation of ABG analyzer–derived serum electrolyte values represents a crucial quality initiative for optimizing patient care.

ABG analyzers provide rapid electrolyte results with accuracy comparable to central laboratory methods within the physiological range [13]. Studies have compared ABG and automated analyzers for Na^+^ and K^+^ measurement [11, 14] and other electrolytes including chloride [14].

Pediatric patients are vulnerable to electrolyte imbalances, which can cause critical states. Serum versus plasma concentrations of Na^+^ and K^+^ ions have been compared to reduce the number of needle punctures required in children admitted to the ICU [5, 15]. However, it is important to note that the present study population consisted exclusively of adult patients (≥ 18 years) and pediatric patients were excluded from our study cohort. Therefore, direct comparisons between our adult population findings and the pediatric literature should be interpreted with caution, as age‐related physiological differences may influence electrolyte measurements and the agreement between different analytical methods. Future research in the pediatric population within our local setting would be valuable to address this gap.

This study has yielded intriguing insights into the measurement of serum electrolyte levels through two distinct methodologies, namely, the ABG analysis as a representation of POCT and the ISE analysis as the gold standard central laboratory approach. A statistically significant difference was observed between the K^+^ values measured by the ABG analyzer and the ISE analyzer (*t*‐test: *p* = 0.024, < 0.05). However, despite this statistical discrepancy, both methods exhibited a strong positive correlation, with a correlation coefficient (*r*) of 0.905 and an *r*‐squared value (*r* ^2^) of 0.819, both of which were significant at a 0.01 significance level.

While assessing the Na^+^ values obtained via the ABG analyzer and the ISE analyzer, no statistically significant difference was observed (*p* value = 0.275, with *p* > 0.05 at a 95% confidence interval). This finding is further supported by a strong positive linear relationship, as evidenced by a correlation coefficient (*r*) of 0.897 and an *r*‐squared value (*r* ^2^) of 0.805, both significant at the 0.01 significance level.

The Bland–Altman analysis further confirmed acceptable agreement between the two methods. For Na^+^, the mean bias was minimal (0.57 mmol/L) with 95% LoAs of −7.69 to 8.83 mmol/L. For K^+^, the mean bias was −0.13 mmol/L with 95% LoAs of −1.04 to 0.78 mmol/L. These ranges are within clinically acceptable limits, supporting the interchangeability of the two methods in routine clinical practice.

Both Na^+^ values and K^+^ values did not have a clinically significant difference when measured using the ABG analyzer and ISE analyzer (Tables 1 and 2) according to the currently practiced clinical standards [6].

The difference between whole blood electrolyte concentrations and serum electrolyte concentrations had been defined earlier [16, 17]. Previous studies have also investigated the evaluation of POCT devices for the measurement of electrolyte concentrations. However, the findings of our study deviated from those of previous investigations. Specifically, our study results revealed that Na^+^ values were in agreement when using both the ABG analyzer and the ISE analyzer. In contrast, there was a statistically significant difference in K^+^ values between the two methods.

Some previous studies have stated that there was no significant difference in K^+^ values when measured using the ABG analyzer and ISE analyzer. Mean K^+^ values were compared when deriving this conclusion. Here, the agreement between the two methods was also acceptable. However, there was a significant difference in mean Na^+^ values, and the agreement between the two methods was also unacceptable [11].

In contrast, there were significant differences in Na^+^ and K^+^ concentrations when they were measured using an ABG analyzer, as the POCT and autoanalyzer in the central laboratory in studies carried out earlier. The agreement between the two methods was unacceptable [18].

Studies with contrasting findings to our results provide important context for interpretation. Gupta et al., in their study published in the Indian Journal of Critical Care Medicine, reported clinically significant differences between ABG and central laboratory electrolyte measurements, particularly in critically ill patients with severe metabolic derangements [19]. Their findings suggested that ABG‐derived electrolyte values may not be fully interchangeable with laboratory values in all clinical scenarios. Similarly, other investigators have reported variable agreement between POCT and central laboratory methods, with some studies showing unacceptable bias for both Na^+^ and K^+^ measurements [20, 21].

These divergent findings across studies highlight the complexity of comparing electrolyte measurements between different analytical platforms. Several methodological factors may contribute to these variations, including differences in patient populations (severity of illness and presence of specific metabolic conditions), sample types (arterial vs. venous blood), timing of sample collection, analyzer models and calibration protocols, and quality control practices. The heterogeneity in study designs and clinical contexts underscores the importance of institution‐specific validation studies.

The deviations between this study′s results from that of the previous study may be due to many reasons. First and most importantly, the same arterial blood sample was used in both ABG and ISE analyzers. The only difference was plasma being separated before the analysis in the ISE analyzer, whereas in many previous studies, arterial blood was used to perform arterial blood gas analysis and venous blood for the ISE analyzers. The difference in sites of blood taken and the matrix difference can also make deviations from those studies [1, 18, 22].

### 4.1. Potential Explanations for the K^+^ Difference

The statistically significant difference observed in K^+^ measurements between ABG and ISE methods (mean difference −0.13 mmol/L, *p* = 0.024) warrants consideration of several potential contributing factors:

Heparin interference: Lithium heparin was used as the anticoagulant in ABG syringes. Although manufacturer specifications indicate minimal interference with K^+^ measurements, trace effects on K^+^ readings cannot be entirely excluded, particularly at the extremes of the reference range.

Plasma–serum matrix variation: ABG analyzers measure whole blood K^+^ using direct ISE methodology, whereas central laboratory ISE analyzers measure plasma K^+^ after centrifugation. Small but measurable differences between whole blood and plasma K^+^ concentrations are well‐documented, typically ranging from 0.1 to 0.3 mmol/L due to cellular K^+^ content and distribution.

Sample handling and timing: Despite immediate processing protocols, the minimal time delay between ABG analysis (performed immediately) and ISE analysis (after centrifugation) could potentially contribute to cellular K^+^ release, particularly in samples with elevated platelet or leukocyte counts.

Analytical variation: The observed mean difference of 0.13 mmol/L falls within the combined analytical imprecision of both methods. When considering the coefficient of variation (CV) for both ABG‐ and ISE‐K^+^ measurements (typically 2%–3%), the observed difference is consistent with expected analytical variability.

Despite the statistical significance, the clinical significance remained minimal (Cohen′s *d*ES = 0.157, indicating a small effect), and the Bland–Altman analysis demonstrated acceptable LoAs (−1.04 to 0.78 mmol/L), well within clinically acceptable ranges for K^+^ measurement. Therefore, these factors likely explain the statistical significance while not compromising clinical utility.

Different patient conditions can also make significant differences in electrolyte concentrations [23, 24]. A study concluded that in conditions of hypoproteinemia, direct ISE and indirect ISE results were not compatible. There, the indirect ISE values for both Na^+^ and K^+^ concentrations were high compared to the direct ISE [12]. Therefore, it is vital to consider the clinical history of the patients before arriving at any conclusions. A future study can be carried out correlating the patient history.

The classification of samples highlighted some important views (Tables 1 and 2). There was an obvious skewness in the sample result distribution. For K^+^ values, this was a positive skewness, and it was comparatively lower. There was a high negative skewness in the Na^+^ value distribution, where 51/67 (76.12%) cases were hyponatremic. For K^+^ values, only 44.78% of hypokalemic cases were found. The skewness of the sample may have contributed to the statistical deviation of this study′s results.

As K^+^ values had a statistically significant difference between the two methods, it is vital to find whether this difference can make a clinically significant difference according to currently practiced clinical categories. However, the number of samples under each category was not adequate to derive such a conclusion in this study (Tables 1 and 2). A future study can be carried out by increasing the sample size under each category, and statistical tools (mean, standard deviation, and *p* value) can be used to evaluate the study results.

A correction factor can be derived if the study reveals that the values obtained under each category are found to be clinically significant. This will enhance the quality of patient management.

### 4.2. Study Limitations

This study has several limitations that should be acknowledged.

Distributional skewness and limited external validity: The dataset exhibits pronounced distributional skewness, with 76.1% of patients presenting with hyponatremia (mean K^+^ ~130 mmol/L). This imbalance means our analysis predominantly reflects ABG analyzer performance within the hyponatremic range, while its accuracy across normonatremic and especially hypernatremic ranges remains inadequately tested. This skewed distribution, while reflecting the real‐world clinical pattern in our emergency setting, significantly limits the external validity and generalizability of our findings. Future research should include a more balanced distribution across all Na^+^ categories to ensure broader applicability and to assess analyzer performance across the full clinical spectrum.

Single‐center design: This study was conducted at a single institution (THK, ETU), which limits the generalizability of findings. Results may not be directly applicable to other institutions with different patient populations, different analyzer models, varying quality control practices, or different preanalytical procedures.

Limited sample size: The sample size of 67 patients, while adequate for overall correlation analysis, restricts statistical power for subgroup analyses within individual electrolyte categories. Particularly, the small number of hypernatremic (*n* = 2) and hyperkalemic (*n* = 6) cases limits robust statistical evaluation within these clinically important subgroups.

Cross‐sectional design: The cross‐sectional nature of this study provides a snapshot assessment but cannot evaluate longitudinal performance, temporal stability of agreement, or the impact of analyzer calibration drift over time.

Emergency setting focus: Results are most applicable to the acute emergency care setting where critically ill patients present with diverse and often severe electrolyte disturbances. Findings may differ in routine laboratory settings, outpatient clinics, or other clinical contexts with different patient acuity levels.

### 4.3. Future Directions

To address these limitations, we recommend multicenter validation studies across diverse clinical settings, larger sample sizes with stratified sampling to ensure adequate representation across all electrolyte categories, prospective longitudinal studies to assess temporal stability, studies incorporating diverse patient populations and clinical conditions, and investigation of analyzer performance in specific patient subgroups (e.g., those with hypoproteinemia, severe metabolic acidosis, or extreme electrolyte derangements).

## 5. Conclusion

Na^+^ concentrations exhibited no statistically significant difference (*p* > 0.05 at the 95% significance level), while K^+^ concentrations demonstrated statistical significance (*p* < 0.05 at the same level of significance) between the ABG and ISE analyses. However, the observed disparities in the concentrations of both parameters were of limited clinical significance, as evidenced by the ES for Na^+^ (ES = 0.062) and K^+^ (ES = 0.157) being less than 0.2, and Bland–Altman analysis demonstrating acceptable LoAs for both analytes.

Therefore, clinicians can confidently rely on the ABG analyzer as a dependable tool for measuring Na^+^ and K^+^ values in the management of critically ill patients. However, these conclusions are most robust for the hyponatremic range, which comprised the majority of our study population. Further validation across a more balanced distribution of electrolyte concentrations would strengthen the generalizability of these findings.

## Author Contributions

Dr. Madhusha Gonapaladeniya was involved in study design and proposal writing, sample and data collection, laboratory work, data analysis and interpretation, and writing the manuscript. Dr. Indika Siriwardana and Dr. Bolonghoge Dayanath designed the study and were involved in proposal writing, data analysis, and writing the manuscript. Dr. Harith Wickramasekara was involved in proposal writing, research questions, data analysis, and writing the manuscript.

## Funding

No funding was received for this manuscript.

## Disclosure

All authors read and approved the final manuscript.

## Ethics Statement

Ethical approval for the study was granted by the Ethics Review Committees of the Faculty of Medicine, University of Ruhuna, and the Teaching Hospital Karapitiya before the initiation of the study. Written informed consent was obtained from each patient or their guardian (if the patient was not in a state to provide voluntary consent due to being unconscious/semiconscious) before enrollment for the study, in accordance with the ethical principles of the Declaration of Helsinki. The patient samples were numerically coded and processed anonymously. The data were stored in a database, and use was restricted for this analysis.

## Conflicts of Interest

The authors declare no conflicts of interest.

## Data Availability

Data will be available upon reasonable request.
